# Development of Therapeutic RNA Manipulation for Muscular Dystrophy

**DOI:** 10.3389/fgeed.2022.863651

**Published:** 2022-05-10

**Authors:** Saifullah, Norio Motohashi, Toshifumi Tsukahara, Yoshitsugu Aoki

**Affiliations:** ^1^ Department of Molecular Therapy, National Institute of Neuroscience, National Center of Neurology and Psychiatry (NCNP), Tokyo, Japan; ^2^ Area of Bioscience and Biotechnology, School of Materials Science, Japan Advanced Institute of Science and Technology (JAIST), Ishikawa, Japan; ^3^ Division of Transdisciplinary Science, Japan Advanced Institute of Science and Technology (JAIST), Ishikawa, Japan

**Keywords:** RNA engineering, antisense oligonucleotides, DMD, exon skipping, molecular therapy, RNA editing therapy

## Abstract

Approval of therapeutic RNA molecules, including RNA vaccines, has paved the way for next-generation treatment strategies for various diseases. Oligonucleotide-based therapeutics hold particular promise for treating incurable muscular dystrophies, including Duchenne muscular dystrophy (DMD). DMD is a severe monogenic disease triggered by deletions, duplications, or point mutations in the *DMD* gene, which encodes a membrane-linked cytoskeletal protein to protect muscle fibers from contraction-induced injury. Patients with DMD inevitably succumb to muscle degeneration and atrophy early in life, leading to premature death from cardiac and respiratory failure. Thus far, the disease has thwarted all curative strategies. Transcriptomic manipulation, employing exon skipping using antisense oligonucleotides (ASO), has made significant progress in the search for DMD therapeutics. Several exon-skipping drugs employing RNA manipulation technology have been approved by regulatory agencies and have shown promise in clinical trials. This review summarizes recent scientific and clinical progress of ASO and other novel RNA manipulations, including RNA-based editing using MS2 coat protein-conjugated adenosine deaminase acting on the RNA (MCP-ADAR) system illustrating the efficacy and limitations of therapies to restore dystrophin. Perhaps lessons from this review will encourage the application of RNA-editing therapy to other neuromuscular disorders.

## Graphical Abstract

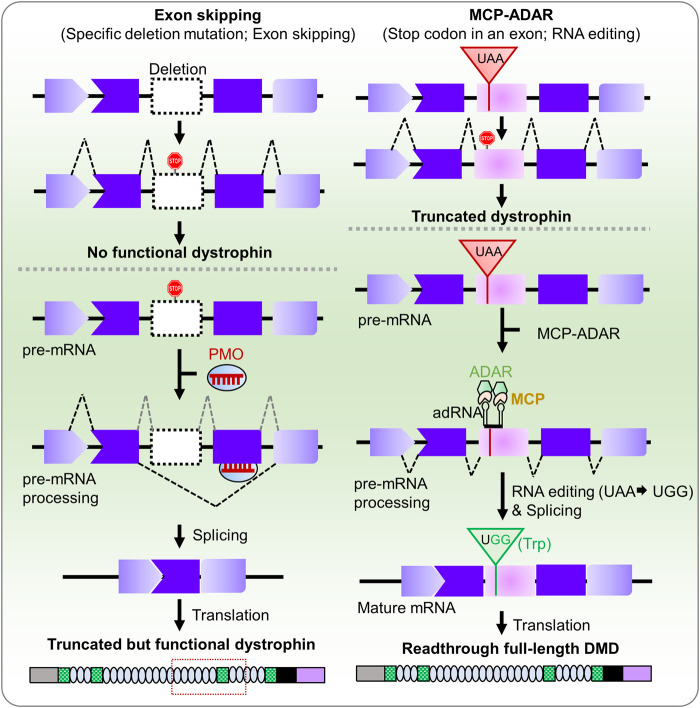

## Introduction

Pharmaceuticals are small compounds that bind to pockets of target proteins, including receptors, enzymes, or other proteins, commonly as antagonists that control a target disease. It is estimated that 3,000 of 20,000 human proteins are targeted by conventional therapeutics (Rodgers et al., 2018). Surprisingly, approved drugs are directed at only 667 human proteins as of 2017 (Santos et al., 2017); thus, the overwhelming majority of human proteins remain untargeted. It is necessary to look beyond the conventional drug approach to tap this unexplored potential. Therapeutic RNA molecules are a promising treatment paradigm because they target disease-causing transcripts in a Watson-Crick base-pairing manner, leading to more precise and tailored medicine for various life-threatening disorders. Therapeutic RNAs primarily represent antisense oligonucleotides (ASOs) (Aoki et al., 2010), small interfering RNAs (siRNAs) (Adams et al., 2018), mRNAs (Polack et al., 2020) to short guide RNAs for the MCP-ADAR system (Azad et al., 2017) or CRISPR/Cas system (Jinek et al., 2012; Saifullah et al., 2020). Very recently, the field has achieved some success with the accreditation of COVID-19 mRNA vaccines for emergency use (Baden et al., 2020; Polack et al., 2020) and approval of two siRNA-based drugs, patisiran and givosiran, for genetic diseases (Adams et al., 2018; Balwani et al., 2020). This clinical pipeline has paved the way for more widespread development of therapeutic RNAs.

French neurologist, Guillaume B. A. Duchenne, first reported a patient with a neuromuscular complication in 1869, and it was later termed DMD (Darras et al., 1993-2021; O'Brien and Kunkel, 2001). Patients with DMD lose the ability to walk at an early age, followed by cardiac and respiratory failure, and eventually death, typically by their 30s (Hanson et al., 2021). DMD is caused by a mutation in the *DMD* gene located at Xp21.2–p21.1 on the X chromosome that affects one out of 3,600–5,000 boys worldwide (Aoki and Wood, 2021; Hanson et al., 2021). Among affected individuals, two-thirds of patients inherit mutated *DMD* from a heterozygous mother who is unaware that she is a carrier, whereas the remaining one-third of cases are acquired *DMD* mutations (Aoki and Wood, 2021). DMD is the largest human gene (∼2.3 mega-bases), comprising 79 coding sequences (exons) with conserved splicing patterns among vertebrates (Dzierlega and Yokota, 2020; Hanson et al., 2021). This conserved splicing pattern enables researchers to apply what they learn from animal models to humans.

The *DMD* gene encodes dystrophin, a subsarcolemmal, rod-shaped protein (427 kDa) that is required for muscle-fiber integrity and protection from contraction-induced injury (Verhaart and Aartsma-Rus, 2019). In the absence of dystrophin, muscle fibers become injured during muscle contraction, leading to progressive muscle wasting, subsequent loss of the ability to walk, and respiratory and cardiac impairments (Mercuri and Muntoni, 2013; Verhaart and Aartsma-Rus, 2019). The molecular basis for the development of DMD includes over 7,000 diverse mutations (Bladen et al., 2015). The most frequent mutation (60–70%) is the deletion of one or more exons, leading to a loss of dystrophin (Olson, 2021). Others, including nonsense mutations, genomic rearrangements, and duplications, are less frequent (Dzierlega and Yokota, 2020). Exon deletions from DMD are typically clustered in two specific “hotspots,” including exons 2–20 and 43–55 (Juan-Mateu et al., 2015). Becker muscular dystrophy (BMD) is a less severe version of the disease phenotype, resulting from a protein, which though truncated, still possesses both the critical N- and C-terminal binding domains, despite lacking some internal rod domains (Dowling et al., 2021). This truncated dystrophin results in a less severe phenotype, with later onset and slower progression of symptoms than DMD (Dowling et al., 2021). For this reason, one of the most common therapeutic strategies for DMD is the restoration of BMD-like dystrophin.

To date, the potency of approved therapies for DMD is very limited, and there is presently no medicine despite extraordinary research efforts. Corticosteroids are commonly used for patients with DMD. These have adverse side effects and minimal efficacy (Drachman et al., 1974; Manzur et al., 2008). Significant efforts have been made to develop therapeutic RNAs to restore functional dystrophin in patients with DMD. Among them, exon skipping using synthetic ASO is currently the leading therapeutic approach for DMD (Komaki et al., 2018; Frank et al., 2020; Wagner et al., 2021). Splice-switching is used to restore the open reading frame (ORF) damaged by out-of-frame deletions. Other strategies include viral vectors expressing recombinant dystrophin (Duan, 2018), cell-based therapy (Morgan et al., 1990; Garcia et al., 2018), exon excision, or mutation repair using CRISPR/Cas9 (Ryu et al., 2018; Nelson et al., 2019). In addition, an emerging novel approach is the restoration of a read-through codon from a nonsense mutation in the *DMD* gene using the MCP-ADAR system (Katrekar et al., 2019).

This review describes clinical progress and state-of-the-art therapeutic RNA manipulation using ASO and other RNA-mediated approaches to treat DMD, and it discusses limitations on their successful implementation in patients.

### Therapeutic Modalities for Patients With DMD

The most common drugs for DMD patients are corticosteroids. In addition, many other drug classes have been used to treat patients with DMD. Broadly, all drugs can be categorized into two major groups. One is pharmacotherapy, using drugs to target downstream pathological mechanisms (Verhaart and Aartsma-Rus, 2019). Pharmaceuticals employed various modalities, including anti-inflammatory, anti-fibrotic, muscle growth and regeneration, calcium homeostasis, vasodilation, cardiomyopathy, osteoporosis, and mitochondrial drugs (Verhaart and Aartsma-Rus, 2019) (Refer to review). However, none of these drugs targets the underlying cause of the disorder—the lack of dystrophin.

The other group of drugs consists of gene and cell therapeutics, which usually target the restoration of functional dystrophin. As conventional drugs are not effective for the treatment of DMD, this strategy is becoming a more common alternative. This group comprises DNA-editing therapy (CRISPR-Cas9; usually exon manipulation) (Long et al., 2014; Li et al., 2015; McTague et al., 2021), gene-addition therapy (microdystrophin with the AAV vector) (Yoshimura et al., 2004; Duan, 2018), RNA-manipulation therapy (Eteplirsen, Golodirsen, Viltolarsen, Casimersen, ASO-cocktail therapy, and RNA-editing therapy), and cell therapy (Motohashi et al., 2019; Yao et al., 2021). Since the lack of functional dystrophin causes DMD, restoring dystrophin is an apparent therapeutic strategy. However, DMD poses several hurdles to gene and cell therapies. First, the target tissue is muscles, which account for 30–40% of body mass (Sartori et al., 2021). The human body has over 500 skeletal muscles, and typically all are affected by DMD (Verhaart and Aartsma-Rus, 2019). Second, muscle fibers are enclosed by a layer of connective tissues that inhibits the delivery of target vectors or stem cells (Verhaart and Aartsma-Rus, 2019). Third, the target gene is *DMD* which encodes one of the largest proteins in the human body (Guiraud et al., 2015). DMD has several thousand known mutations (Sheikh and Yokota, 2020); therefore, it is not easy to design a single therapy to restore it. Fourth, children are affected by DMD early in life and lose muscle mass (Bendixen et al., 2012). Though restoration of dystrophin theoretically slows down or stops the development of DMD, it does not restore muscle tissue that has already been lost. Therefore, early treatment is needed. Another hurdle is an intense inflammation and immune response in dystrophin-deficient muscle cells due to contraction-induced-injury (Rosenberg et al., 2015). The dystrophin inadequacy damages muscle cells and constitutively secrets TLR ligands and ATPs in the extracellular region, triggering an innate immune response (Giordano et al., 2014). On the other hand, proinflammatory cytokines activate adaptive immunity through continuous activation of MHC I and II and subsequent recruitment of T- and B- cells in the vicinity of damaged muscle (Rosenberg et al., 2015; Farini et al., 2021). Thus, the immunological matter is a challenge for the development of DMD gene therapy (Mendell et al., 2010; Vila et al., 2019).

### Therapeutic RNA Manipulation for DMD

Despite the hurdles mentioned above, considerable effort has been devoted to developing gene therapies for patients with DMD. The leading gene therapy strategy is RNA-manipulation therapy, in which an interrupted *DMD* transcript is engineered to restore the function of dystrophin by repairing the translation reading frame. Modes of action, progress, and limitations of RNA-manipulation therapeutic strategies are discussed below.

### Eteplirsen

Eteplirsen was developed by Sarepta Therapeutics, Inc. (Cambridge, MA, United States). Although the United States Food and Drug Administration (FDA) advisory committee voted not to approve eteplirsen in the beginning, the FDA administratively overrode that and gave accelerated approval in 2016 as the first genetic medicine for DMD (FDA, 2016). It is a 30-bp, synthetic, single-stranded, antisense oligonucleotide, modified with phosphorodiamidate morpholino oligomer (PMO) with the sequence 5′-CTCCAACATCAAG GAAGATGGCATTTCTAG-3' (Arechavala-Gomeza et al., 2007; Kole and Krieg, 2015; Mendell et al., 2016). Unlike DNA or RNA, PMO bases are bound to a morpholine moiety, and subunits are linked through neutrally charged phosphorodiamidate (Kole and Krieg, 2015). Eteplirsen is amenable to exon 51 skipping in the *DMD* gene. Eteplirsen is a broad-spectrum drug covering ∼20.5% of deletion mutations in this gene, which account for ∼13% of all DMD patients (Bladen et al., 2015).

**FIGURE 1:**
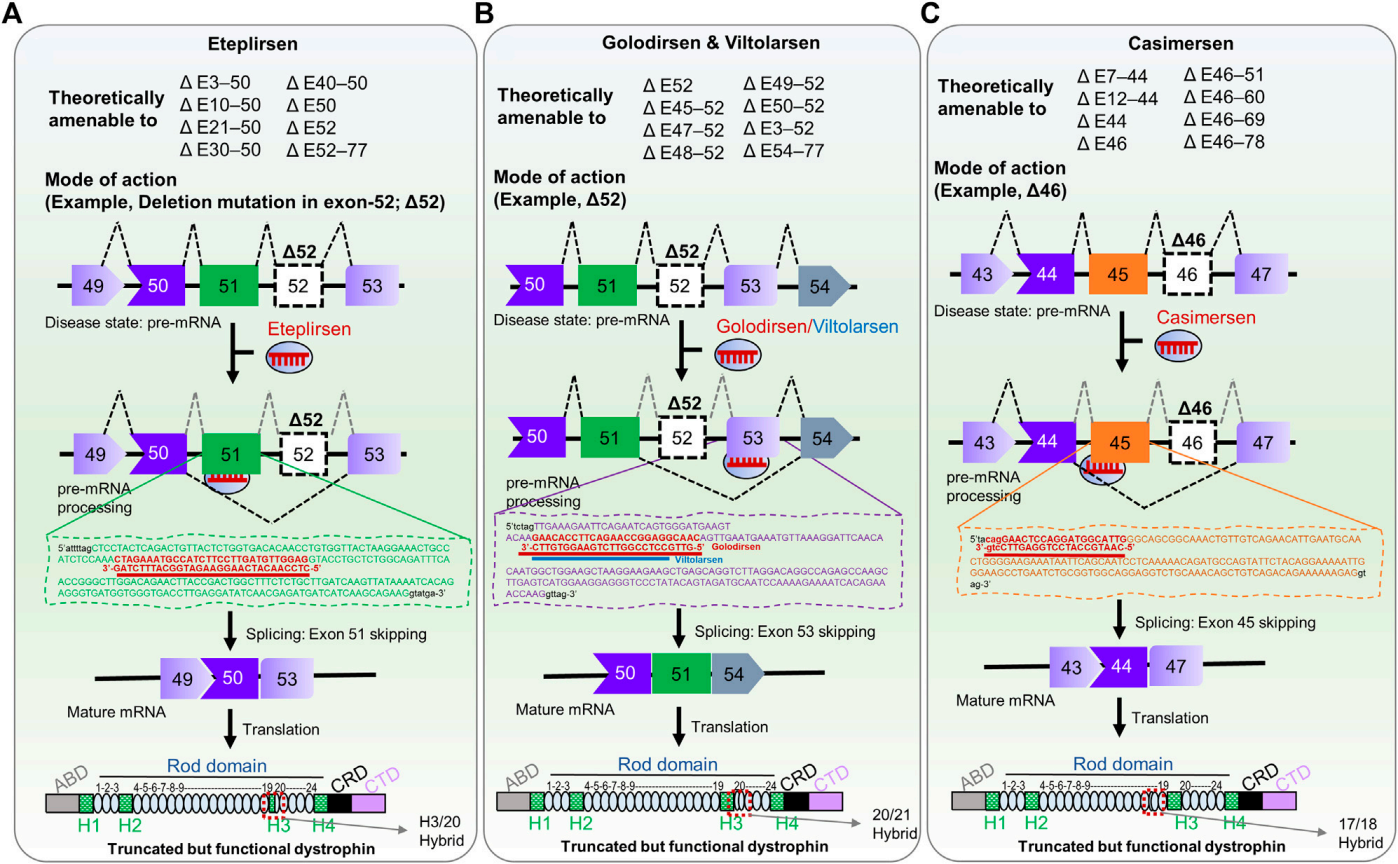
FDA-approved RNA therapies for patients with confirmed, specific *DMD* mutations. **(A)** Eteplirsen, a morpholino ASO that promotes exon 51-skipping in patients with DMD. **(B)** Golodirsen & Viltolarsen, morpholino ASOs that causes exon 53-skipping in DMD patients. **(C)** Casimersen, is a morpholino oligonucleotide that skips exon 45 in patients with DMD. The potential deletion mutations that could be responsive to each therapy are shown in upper panels (Aartsma-Rus et al., 2009; Fletcher et al., 2010); Δ, deletion mutation; E, exon; the number indicates exon number of DMD gene. The mechanism of action of each therapy is shown in the lower panel as an example of deletion mutation exon 52 (Δ52) and deletion mutation exon 46 (Δ46). The ultimate fate of exon skipping in dystrophin structure is shown in each panel; ABD, Actin binding domain; H1-H4, Hinge domains; Rod domain contains 24 domains; CRD, Cysteine-rich domain; CTD, C-terminal domain. The exon 51/52 skipping, exon 52/53 skipping, and 45/46 skipping resulted in the H3-Rod20 hybrid (H3/20 as indicated), Rod 20/21 hybrid, and Rod 17/18 hybrid dystrophin, respectively.

Eteplirsen hybridizes with the complementary sequence of exon 51, leading the spliceosome to skip exon 51 in the ORF during pre-mRNA splicing, ultimately restoring functional, albeit shortened, dystrophin (Figure 1A) (Lim et al., 2017). The efficacy of eteplirsen was examined in an engineered *Dmd* KO mouse carrying the human *DMD* gene (Echigoya et al., 2017a). Based on successful exon 51 skipping in the mouse model, this medicine has progressed in clinical trials, acquiring an efficacy and safety profile. In the trial (NCT01396239), 7- to 13-year-old patients who were treated with 30 mg/kg/week of an eteplirsen analog showed a 23% restoration of dystrophin-positive myofibers based on immunohistochemistry (IHC) result (*p* ≤ 0.002) at 24 weeks, in comparison with the placebo control group (Mendell et al., 2013). The same dose cohort showed a greater restoration efficiency of dystrophin (52%) at 48 weeks. Since the FDA found the IHC method questionable, Western blot-based dystrophin expression from muscle biopsies had shown average 0.44% restoration compared to healthy individuals. A similar degree (0.22%–0.32%) of restoration was observed at 48 weeks of 30 mg/kg/week-treatment regimen in 13 patients in the NCT02255552 intervention study. However, there was no significant difference in the 6-min walk test (6 MWT) result. The higher-dose cohort (50 mg/kg/week) showed considerable 6-min walking capacity with respect to the control group (*p* ≤ 0.001).

NCT01396239 and other eteplirsen clinical trials found only a delay in disease development rather than any substantial improvement in disease symptoms, with relatively low efficiency, based on muscle biopsy results (Cirak et al., 2011; Mendell et al., 2016). One concern was that 2 of 12 patients lost their walking capacity (Dzierlega and Yokota, 2020). Factors possibly associated with this poor outcome include a) poorly selected ASO sequences, based on a bioinformatic analysis (Echigoya et al., 2017a), b) the neutral charge nature of PMO, which may reduce uptake of ASO due to rapid clearance (Lim et al., 2017), c) a connective tissue barrier that prevented the drug from reaching target muscle cells (Verhaart and Aartsma-Rus, 2019), d) difficulties in crossing membranes (Dzierlega and Yokota, 2020), and e) heterogeneity of subject age (since age may alter severity and drug response). In 2018, the European medical agency (EMA) declined to market eteplirsen to treat patients with DMD for lack of sufficient efficacy (Aartsma-Rus and Goemans, 2019).

### Golodirsen

In 2019, the FDA granted accelerated approval for another genetic medicine called Golodirsen, developed by Sarepta Therapeutics, Inc., to treat patients with confirmed mutations amenable to exon 53 skipping of *DMD* (FDA, 2019). Golodirsen is a synthetic, 25-bp PMO (shorter than eteplirsen) with the sequence 5′-GTT​GCC​TCC​GGT​TCT​GAA​GGT​GTT​C-3' (Dzierlega and Yokota, 2020). Golodirsen is useful for fewer patients with DMD mutations (∼8%) than eteplirsen (Frank et al., 2020).

Golodirsen binds to exon 53 of *DMD* pre-mRNA via Watson-Crick base pairing and restores the reading frame of the transcript in patients with *DMD* mutations, producing a truncated yet functional dystrophin (Figure 1B). In Phase I/II clinical trials (NCT02310906), patients treated with 30 mg/kg/week of golodirsen increased dystrophin-positive fibers from 0.10 to 1.02% (∼16-fold) compared to baseline at 48 weeks (Frank et al., 2020). After the treatment, the *de novo* dystrophin was confirmed at the sarcolemma with a substantial increase of 10.47% (Frank et al., 2020). However, clinical benefits have yet to be explored in Phase III clinical trial (FDA, 2019). This study aims to investigate the clinical benefit of golodirsen regarding motor functions (NCT02500381). In this double-blind, placebo-controlled intervention, 30 mg/kg/week of golodirsen will be administrated through intravenous infusion for up to 96 weeks to >200 participants. The estimated completion time of this trial is April 2024.

Muscle biopsies showed 1% dystrophin restoration after approximately a year of systemic dose administration, which is insufficient to yield substantial clinical benefits. Better sequence design, chemical modification, or drug delivery is essential for clinical benefits.

### Viltolarsen

Viltolarsen (code name: NS-065/NCNP-01) is a single-stranded, antisense PMO complementary to exon 53 of *DMD,* developed jointly by Nippon Shinyaku Co., Ltd. and the National Center of Neurology and Psychiatry (NCNP), Tokyo (Watanabe et al., 2018; Clemens et al., 2020). Viltolarsen is 4 bp (5′-GTTG-3′) shorter at the 5′ end than golodirsen, with the same target position (5′-CCT​CCG​GTT​CTG​AAG​GTG​TTC-3′) (Dzierlega and Yokota, 2020). Targeting exon 53 with exon-skipping therapy can be used for patients with deletion mutations of exons 52, 45–52, 47–52, 48–52, 49–52, or exons 50–52 (Watanabe et al., 2018). As such, viltolarsen could potentially be used to treat 10.1% of all DMD patients (Watanabe et al., 2018).

Like golodirsen, viltolarsen exhibits a similar mode of action for skipping exon 53 (Figure 1B). Watanabe et al. (2018) extensively investigated the position, length, and efficiency of PMOs targeting exon 53 in DMD patient-derived cells, and viltolarsen proved the best candidate, based on dystrophin-restoration efficiency (87.1%). It now advances to Phase I clinical trials for safety, tolerability, and pharmacokinetic profiling (Komaki et al., 2018). In a Phase I trial, 1.25, 5, or 20 mg/kg/week of viltolarsen was administered intravenously for 12 weeks, with no severe adverse reactions. However, there were 72 concerns, including an elevation of interleukin and N-acetyl-β-D-glucosaminidase (NAG) concentrations, proteinuria, albuminuria, and anemia (Komaki et al., 2018). Viltolarsen caused no significant renal toxicity or immunogenicity in any patients, suggesting its safety and tolerability for patients, and its efficacy was dose-dependent. Dystrophin-positive fibers were detected in 2/10 patients. The low efficacy observed in this Phase I trial may be due to lower doses and shorter durations. Surprisingly, exon skipping was confirmed in seven out of ten patients, with one patient having 47.5% skipped exons (Komaki et al., 2018).

In a Phase II intervention involving 16 boys, viltolarsen (NCT02740972) was administered intravenously at 40 mg/kg/week and 80 mg/kg/week doses for up to 24 weeks (Clemens et al., 2020). Compared to baseline, the levels of *de novo* dystrophin increased on average 5.7% and 5.9%, respectively. The 6 MWT showed substantial progress in which ambulation of the treatment group was ∼29 m compared to -65.3 m for the control group, as reported by Clemens et al., 2020. Contraindications of viltolarsen included injection site reaction, fever, and upper respiratory tract infection, with no serious adverse events or deaths. Notably, dystrophin levels induced by viltolarsen are the highest reported in clinical studies of exon-skipping therapies to date (Charleston et al., 2018; Muntoni et al., 2018). Based on its excellent safety, tolerability, and efficacy profile, the Pharmaceuticals and Medical Devices Agency (PMDA), Japan, and the United States FDA granted early conditional approval in March 2020 and accelerated approval of viltolarsen in August 2020 for DMD patients with confirmed mutations amenable to exon-53 skipping (FDA, 2020; PMDA, 2020). However, further clinical advances, suggested by both regulatory agencies, need to be investigated in Phase III trials. Accordingly, a Phase III trial (NCT04768062) is underway to profile the safety and efficiency of viltolarsen in 74 DMD patients. Participants will receive 80 mg/kg/week of viltolarsen intravenously for up to 96 weeks. The expected completion date is in June 2026.

### Casimersen

The FDA granted another therapeutic RNA drug called Casimersen (Amondys 45) in February 2021 as an accelerated approval for treatment of DMD in patients who harbor confirmed mutations amenable to exon 45 skipping (Figure 1C) (FDA, 2021). The base sequence of Casimersen is 5′- CAA​TGC​CAT​CCT​GGA​GTT​CCT​G–3'. Casimersen will cover 8% of all DMD patients (Aartsma-Rus et al., 2009). In Phase I/II double-blind, dose titration intervention, 30 mg/kg/week of casimersen were administered intravenously to participants 7–20 years of age. It showed a remarkable restoration of dystrophin level compared to placebo controls. Adverse effects included respiratory tract infections, fever, cough, throat pain, joint pain, and headache (FDA, 2021; Wagner et al., 2021). A Phase III placebo-controlled intervention with the same clinical trial number as golodirsen (NCT02500381) is presently underway. This study will collect further evidence on clinical benefits, safety, and efficacy, especially for ambulatory patients.

### PMO Cocktail Therapy

Single-exon-skipping therapy can potentially cover only10% of all DMD patients. Simultaneously targeting multiple exons using a PMO cocktail may be able to treat >90% of all DMD patients (Yokota et al., 2012). Importantly, even patients harboring a single-exon defect can benefit from this PMO cocktail therapy (Yokota et al., 2007). A proof-of-concept study of multiexon skipping was done in an *mdx* mouse model to skip exons 19–25 to restore dystrophin expression lost due to a premature stop codon in exon 23 (Fall et al., 2006). This cocktail therapy consisted of nine PMOs to skip the target exons. The cocktail showed higher efficiency in restoring dystrophin than therapeutics targeting a single PMO. The strategy was also administrated in canine X-linked muscular dystrophy targeting exons 6–8 to restore dystrophin that is lost due to a single-base mutation in intron 6 (Miskew Nichols et al., 2016). Dystrophin-positive fibers were significantly increased compared to the control group. An increase in dystrophin-positive heart muscle and improved walking were observed with no noticeable toxic effects in the cocktail-treated group.

**FIGURE 2:**
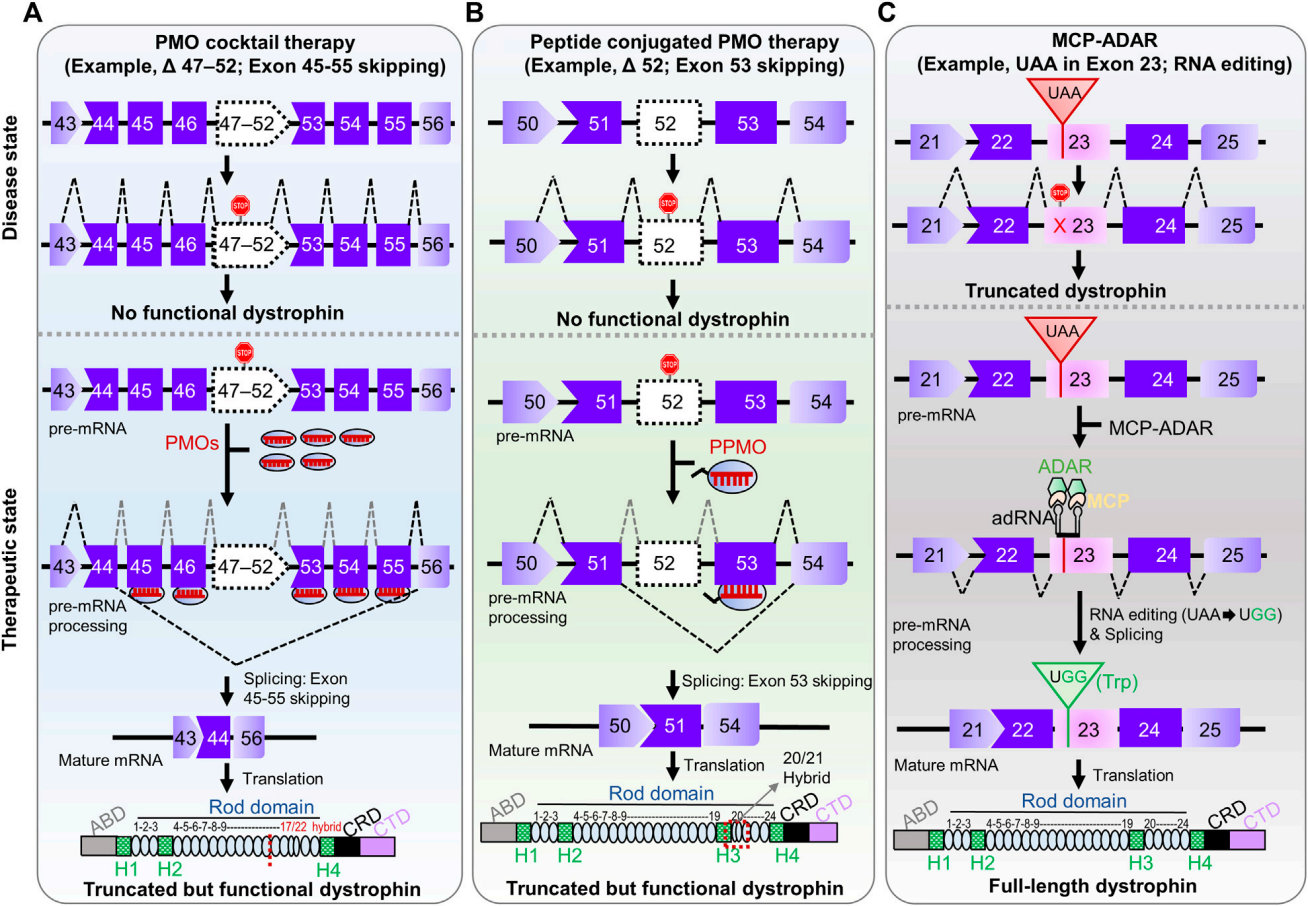
Pre-clinical RNA-manipulation therapies for DMD. **(A)** PMO cocktail therapy contains multiple PMOs complementary to multiple target exons (exon 45–55, a so-called mutation hotspot) amenable to multiple exon-skipping yields truncated but preserved function. **(B)** Peptide-conjugated PMO therapy involves conjugation of a cell-penetration peptide to the morpholino ASO targeting an exon. The ultimate fate of exon skipping in dystrophin structure is shown in each panel; ABD, Actin binding domain; H1-H4, Hinge domains; Rod domain contains 24 domains; CRD, Cysteine-rich domain; CTD, C-terminal domain. **(C)** MCP-ADAR therapeutic strategy uses ADAR enzyme fused to MS2-coat protein to convert a stop codon (UAA) into a read-through codon (UGG; Trp) and produces a full-length dystrophin.

Notably, a pre-clinical proof-of-concept study of exon 45–55 skipping using PMO cocktails targeting a mutation hotspot was executed successfully (Figure 2A) (Aoki et al., 2012). Targeting exon 45–55 using a PMO cocktail potentially covers 63% of deletion mutations and 45% of all DMD patients (Aoki et al., 2012). The efficiency of the PMO cocktails was examined in *H2K*-*mdx52* muscle cells and in *mdx52* mouse models, which lack exon 52. Dystrophin-positive fibers were restored up to 15% in the *in vivo* mouse model with improved muscle strength and no noticeable toxic effect in the treatment group. The effectiveness and safety of the PMO cocktail targeting exon 45–55 were also investigated in a long-term follow-up (16 weeks) study in an *mdx52* mouse model (Echigoya et al., 2015). A significant increase in dystrophin-positive fibers (5–27%) was observed with no adverse effect. Similarly, Echigoya et al. (2019) screened three sets of PMO cocktails in a cell-based assay, using DMD patient-derived cells, and humanized mouse models. This study found a similar degree of dystrophin restoration efficiency (15–22%) in the mouse models (Echigoya et al., 2019). Despite this successful demonstration, PMO cocktail therapy faces challenges moving into clinical studies due to regulatory obstacles (Dzierlega and Yokota, 2020).

### Peptide-Conjugated PMO Therapy

After several clinical trials of morpholino ASOs, it is evident that this strategy faces a challenge relative to efficacy, especially in long-term follow-up studies. One potential strategy would be the development of peptide-conjugated PMO therapy to address this. In this strategy, a short cell-penetrating peptide is conjugated to the morpholino ASO, which substantially improves cellular uptake and potency (Tsoumpra et al., 2019; Tone et al., 2021). In a proof-of-concept study, this therapy restored 40–50% dystrophin expression in *mdx* mice relative to wild-type mice (Jearawiriyapaisarn et al., 2008). Another group found 25–100% dystrophin-positive fibers in skeletal muscle throughout the body, with no severe adverse effect in an *in vivo* study (Yin et al., 2008).

The high potency of peptide-conjugated PMO therapy has now advanced to clinical trials. Sarepta Therapeutics, Inc. started a Phase I clinical trial of a peptide-conjugated morpholino ASO (SRP-5051) able to skip exon 51 (NCT03375255). The purpose of this study was to profile the safety, acceptability, and pharmacokinetics of a single-dose intravenous infusion (IV) in 12-year or older patients with DMD. Recently, the same pharmaceutical company recruited 60 subjects age 4 or older in Phase I/II clinical study (NCT03675126) to evaluate the safety, tolerability, and doses of SRP-5051 in patients with DMD amenable to skipping exon 51.

The main hurdle for future clinical intervention for peptide-conjugated ASO therapy is toxicity, including lethargy, weight loss, tubular degeneration in the kidney (Amantana et al., 2007; Wu et al., 2008; Said Hassane et al., 2010). Factors determining the toxicity are duration and frequency of drug administration, drug concentration, the type of conjugating peptide, peptide chemistry, the exon to be skipped, and complement activation due to immunogenicity (Moulton and Moulton, 2010; Echigoya et al., 2017b; Tsoumpra et al., 2019). Notably, the first-generation arginine-rich peptides exerted more robust immune responses than morpholino oligos (Muntoni and Wood, 2011). A complete toxicity and pharmacokinetics profile will be necessary before peptide-conjugated ASO therapy can be approved for routine clinical intervention.

### MCP-ADAR Therapeutic Modality

Stop-codon read-through is a therapeutic modality for DMD that enables the translation of full-length dystrophin from truncated dystrophin caused by a premature stop codon. This strategy is applicable to 10–15% of all patients with DMD (Finkel, 2010). Aminoglycoside antibiotics were initially used, including gentamicin, negamycin, and G-418 (Malik et al., 2010a; Sheikh and Yokota, 2021). The clinical benefit could not be reproduced using gentamicin in an *mdx* mouse model (Malik et al., 2010b). Ataluren (PTC124/Translarna) is a small molecule that selectively induces read-through of premature stop codons but not normal termination codons (Welch et al., 2007). A nonsense-reporter in patient-derived myotubes and mdx mice showed an increase in dystrophin-positive fibers. It was well tolerated, and oral bioavailability increased in clinical trials. However, the FDA rejected ataluren due to insufficient data, while it received conditional approval in Europe, South Korea, and Israel (Landfeldt et al., 2019). In observational clinical studies, ataluren has yielded inconsistent results in skeletal and heart muscles in patients with DMD (Mercuri et al., 2020).

Another novel approach to stop-codon read-through is artificial RNA editing technologies (Stafforst and Schneider, 2012; Montiel-Gonzalez et al., 2013; Cox et al., 2017; Fukuda et al., 2017; Sinnamon et al., 2017; Vallecillo-Viejo et al., 2018). One of the methods is the MCP-ADAR system, which conjugates the deaminase domain of ADAR enzyme to MS2 bacteriophage coat protein (MCP) (Azad et al., 2017; Katrekar et al., 2019). The MCP protein can recognize MS2 RNA containing target guide RNA, enabling it to navigate to the target site (Azad et al., 2019; Bhakta et al., 2019; Tohama et al., 2020). This system translated premature stop codons with 5–27% efficiency in human cells (Azad et al., 2017; Bhakta and Tsukahara, 2021). Thus, the MCP-ADAR system enables translation machinery to substitute a tryptophan codon (UGG) for all three stop codons (UAG, UGA, and UAA) (Bhakta and Tsukahara, 2021). The MCP-ADAR system has also been used for C-to-U base editing by replacing ADAR with APOBEC1 (apolipoprotein B mRNA-editing enzyme, catalytic polypeptide 1) enzyme with 21% efficiency (Bhakta et al., 2020). Recently, the system has been applied to mouse models of ornithine transcarbamylase (OTC) and *Dmd* deficiencies (Katrekar et al., 2019). The results showed 4.6–33% and 3.6% restoration efficiency, respectively. Improvement of this system can be achieved by altering the MS2 stem-loop, the binding site of MS2 coat protein, using gRNAs of different lengths and positions, altering the delivery system [(for example, Adeno Associated Virus (AAV) or Lipid nanoparticle (LNP)], or dose titration of expression vectors. However, there is a concern about immunogenic reactions to MCP from the viewpoint of RNA manipulation therapy in clinical intervention.

### Structural Modification of Therapeutic RNAs

Structural modification of therapeutic RNA is one of the most effective strategies to increase ASO-mediated drug delivery efficiency. Specifically, modification improves ASO pharmacokinetics, biodistribution, and its pharmacodynamics profile (Roberts et al., 2020). In the first era of genetic medicine, the phosphodiester backbone of ASOs was altered to make phosphorothioate, methylphosphonate, and phosphoramidite (Järver et al., 2014; Aoki and Wood, 2021). These modifications are readily tolerated and do not interfere with RNase H activity (Freier and Altmann, 1997; Aoki et al., 2013). One shortcoming of this phosphodiester modification is its low binding capability to target complementary strands (Roberts et al., 2020).

In the second era of ASO modification, three structural alterations were widespread, including 2ʹ-methyl phosphorothioate (2ʹ-OMe) and 2ʹ-methoxyethylene (2ʹ-MOE), 2ʹ-fluoro nucleotides, in which the 2ʹ-hydroxyl group of RNA is substituted, thereby resisting nuclease activity (Tsoumpra et al., 2019; Aoki and Wood, 2021). Increased stability in blood plasma, improved binding activity to the target site, and prolonged drug effects have been observed due to these structural modifications (Southwell et al., 2012; Roberts et al., 2020). One of the genetic medicines, called drisapersen, uses a 2ʹ-OMe modified ASO that targets exon 51 skipping in *DMD*. However, drisapersen could not show sufficient dystrophin-positive fibers and resulted in serious adverse drug effects in a long-term Phase III intervention (Trial number: NCT01480245). This adverse effect in the clinical study led to further structural modifications for ASO therapy.

PMOs, locked nucleic acids (LNAs), peptide nucleic acids (PNAs), tricyclo DNAs, and ethylene-bridged nucleic acids (ENA) belong to the third generation of structural ASO modifications (Aoki and Wood, 2021). PMOs are the leading ASO for DMD therapy in which the charged deoxyribose ring is substituted with a non-ionic morpholine ring (Iversen, 2001; Moulton, 2017). The current four FDA-approved genetic medicines for DMD patients are PMO-based modifications. The PMO has been the only chemistry to make it through clinical intervention, whereas all others have failed or have not made it passed the pre-clinical stage due to adverse side effects, low binding capacity, or low internalization efficiency (Goemans et al., 2016; Roberts et al., 2020). This modification strategy can bypass nuclease activity with no severe adverse effects; however, their uncharged nature reduces cellular uptake (Warfield et al., 2006; Aoki and Wood, 2021). In line with this consideration, conjugation of a cell-penetrating peptide to PMOs is a current trend to improve cellular uptake. This modification greatly increased cellular uptake and improved its pharmacokinetic profile (Tsoumpra et al., 2019; Tone et al., 2021). It is important to note that both PMO and PNA interact minimally with blood plasma proteins, suggesting that they are quickly excreted in the urine (Roberts et al., 2020). Structural modification of guide RNAs is not necessary for MCP-ADAR therapeutic modality.

### Advantages and Challenges

The field of therapeutic RNA manipulation continues to develop and represents a revolutionary treatment paradigm for many muscular dystrophies, including DMD. The potential benefits of using RNA manipulation rather than DNA manipulation therapy include a) no chance of a permanent error, whereas DNA manipulation could potentially create a new permanent problem (Saifullah et al., 2020; Saifullah et al., 2021) b) since our cellular system expeditiously destroys unused RNA, any miscue generated by RNA manipulation therapy would eventually be discarded, c) mRNAs are most commonly expressed in a tissue-specific manner (Fagerberg et al., 2014), hence, reducing chances of an erroneous transcript, d) it requires a minimal number of tools or machinery that minimizes immunogenic reactions compared to genome editing toolsets. In therapeutic RNA manipulation, the most promising approach is exon skipping for the treatment of DMD. Until January 2022, the FDA has approved four exon-skipping morpholino ASOs. Of those, viltolarsen shows the most promising therapeutic RNA manipulation.

Despite these tremendous advantages, several issues must be addressed for further clinical success of RNA-manipulation therapy. First, how can the efficacy of RNA manipulation therapy be improved for DMD? A precise drug delivery system could be one answer. To address this issue, a cell-penetrating peptide-conjugated PMO is being developed (Tone et al., 2021) and a clinical intervention conducted by Sarepta Therapeutics Inc. is underway. Antibody-conjugated ASO therapy could enhance targeted myofiber uptake, potentially improving efficacy (Hammond et al., 2021). One potential modality would be the addition of a short myofiber-specific nuclear signal peptide (of human origin) to the peptide-conjugated PMO, which theoretically could enhance the nuclear internalization of target ASOs. Since genes associated with drug absorption, dispensation, metabolism, and clearance are polymorphic, efficacy and toxicity of drugs vary among individuals (Jittikoon et al., 2016; Saifullah and Tsukahara, 2018; Saifullah, 2018; Saifullah et al., 2019). Therefore, personalized genetic medicine could enhance drug responses in DMD patients.

Second, how can the toxicity of morpholino ASO be reduced? Though all approved genetic medicines for DMD are well tolerated, there are still toxicity concerns (Komaki et al., 2018; FDA, 2019). One of the reasons is that ASOs generally accumulate in the liver, spleen, kidney, and bone marrow (Aoki and Wood, 2021). Alteration of PMOs or a new naturally occurring candidate would be an option. Lipid nanoparticle (LNP) has been successfully used in COVID-19 vaccines (Baden et al., 2020; Polack et al., 2020), which might be an alternative approach for future DMD therapeutics. Third, how long can therapeutic RNAs be sustained in patients with DMD? This is one of the important drawbacks of RNA-manipulation therapy, as we have learned from the necessity of boosters with COVID-19 mRNA vaccines. Fourth, what would be the ideal age of DMD patients to take genetic medicine? When is it too late? One suggestion is to administer them as early as the disease is diagnosed. This is because all genetic medicines can produce positive fibers but cannot replace muscle that has already been lost due to disease progression. However, clinical evidence is urgently needed for effective treatment.

## Future Prospects and Conclusion

Currently, manipulation of pre-mRNA using exon-skipping therapy is deliberated as the most promising strategy for DMD treatment. Within the last few years, the accreditation of exon-skipping drugs by the FDA and PMDA as a novel therapeutic modality has moved them to the forefront of next-generation genetic medicines for muscular dystrophies, including DMD. The optimization of exon skipping has sparked great interest and can significantly change the lives of the many affected individuals by a wide range of incurable genetic diseases. As the progress continues, we are optimistic about the success of this strategy and are eager to see what the future hold.

While this paper has focused on RNA-manipulation therapy to correct DMD mutations, various exciting genome editing approaches using CRISPR/Cas9 are being developed to repair out-of-frame mutations. Collectively, we are venturing into unprecedented territory for rare genetic neuromuscular medicine, with the hope of treatments for ultrarare incurable diseases. It is worth noting that despite this significant progress, there is still an enormous unmet need to understand pathogenic mechanisms of disease progression better and to enhance drug delivery strategies. Finally, lessons learned from DMD should be applicable to other neuromuscular disorders and numerous life-threatening genetic diseases for which no effective therapy currently exists.
